# A Rare Case of Vaginal Leiomyoma: A Case Report and Review of Literature

**DOI:** 10.7759/cureus.76643

**Published:** 2024-12-30

**Authors:** Alokananda Ray, Sarita Kumari

**Affiliations:** 1 Obstetrics and Gynaecology, Tata Main Hospital, Jamshedpur, IND

**Keywords:** histopathology, imaging modalities, mri, symptoms, tvs, vaginal leiomyoma, vaginal myomectomy

## Abstract

Leiomyomas are benign tumors of the female genital tract, usually arising from the uterus. Vaginal leiomyomas are extremely rare. We describe here a case of vaginal leiomyoma in a 28-year-old unmarried woman who presented with excessive vaginal bleeding and acute retention of urine. Magnetic Resonance Imaging (MRI) helped in diagnosing the vaginal origin of the mass. It was treated by complete excision through the vaginal route and repair of the vaginal defect. Injection adrenaline was used in 1:200000 dilution to identify the tumor extent and create an avascular plane for safe enucleation of the myoma. Post-surgery, the patient recovered well, with good healing of the vaginal wound and complete recovery from voiding difficulty. Histopathology was suggestive of benign vaginal leiomyoma. Thus though rare vaginal leiomyomas should be considered in the differential diagnosis of benign vaginal tumors, they are best identified by imaging modalities like transvaginal ultrasonography (TVS) and MRI. Vaginal myomectomy with repair of the vaginal defect is the most prevalent and conventional method of treatment. The diagnosis is confirmed by histopathological examination of the excised tumor.

## Introduction

Leiomyomas are benign tumors that consist of varying amounts of fibrous tissue and smooth muscle fibers. The uterus is the most common site of leiomyoma, affecting 20-30% of women in the reproductive age group [1]. Vaginal leiomyomas are extremely uncommon, mostly arising from the anterior vaginal wall and less frequently from the posterior and lateral walls of the vagina, with varied clinical presentations [2]. Typically, they are single, well-circumscribed, benign, slowly growing tumors that may or may not be pedunculated [2]. We describe here a case of highly vascular vaginal leiomyoma and its successful management.

## Case presentation

A 28-year-old nulliparous woman presented with foul-smelling vaginal discharge for 10 days and excessive vaginal bleeding with retention of urine for one day. She gave a history of something coming out of the vagina and difficulty in passing urine for the last six months, for which she had not sought any medical attention. Her previous menstrual cycles were regular at 28 to 30 days intervals, lasting for four days with normal flow and without dysmenorrhea. Her previous medical, surgical, personal, and family history were unremarkable, and presently she was not sexually active.

**Figure 1:**
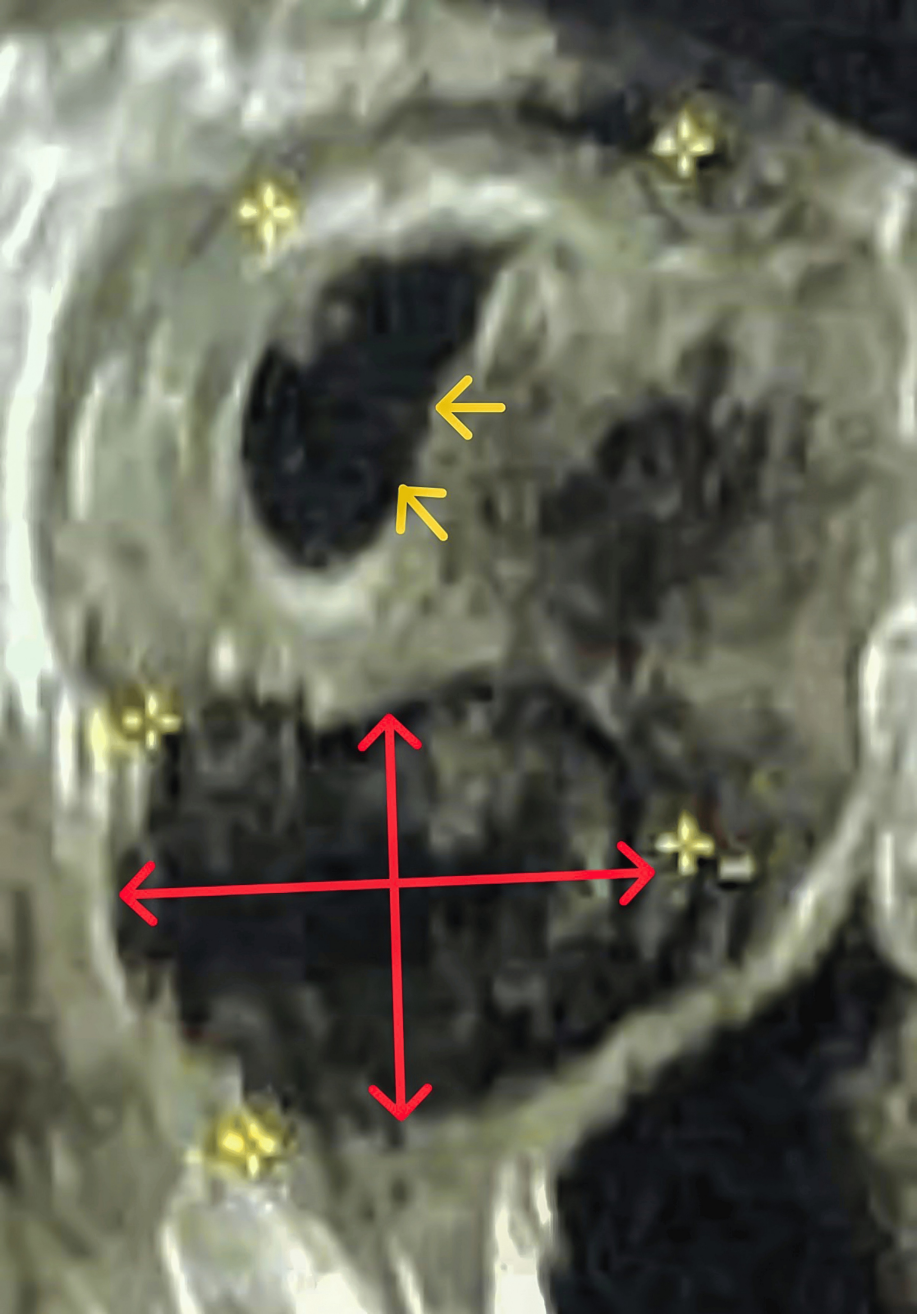
On transvaginal ultrasonography (TVS) a mass was seen in the vagina measuring 5.8x4.2 cm (marked in red). The uterus was normal in size and echo pattern with mild endometrial collection (marked by yellow arrows).

On clinical examination, she had mild pallor, and her vitals were stable. On catheterization of the urinary bladder, about 1000 ml of clear urine was drained. Local examination revealed a bleeding mass of 6x4.5 cm arising from the anterior vaginal wall in the midline, which was soft to firm in consistency with a smooth surface. The cervix was seen separate from the mass and was normal in appearance. On bimanual examination, the uterus was normal in size, anteverted, and mobile, and both the lateral and posterior fornices were free. Examination of all other systems was within normal limits. Other than the detection of anemia (hemoglobin of 9.6gm%, reference range 11.5 gm% to 16gm%), all laboratory investigations of blood and urine (including coagulation profile) were normal. On transvaginal ultrasonography (TVS), a mass was seen in the vagina measuring 5.8x4.2 cm. The uterus and ovaries were normal in size and echo pattern with mild endometrial collection (Figure 1).

**Figure 2:**
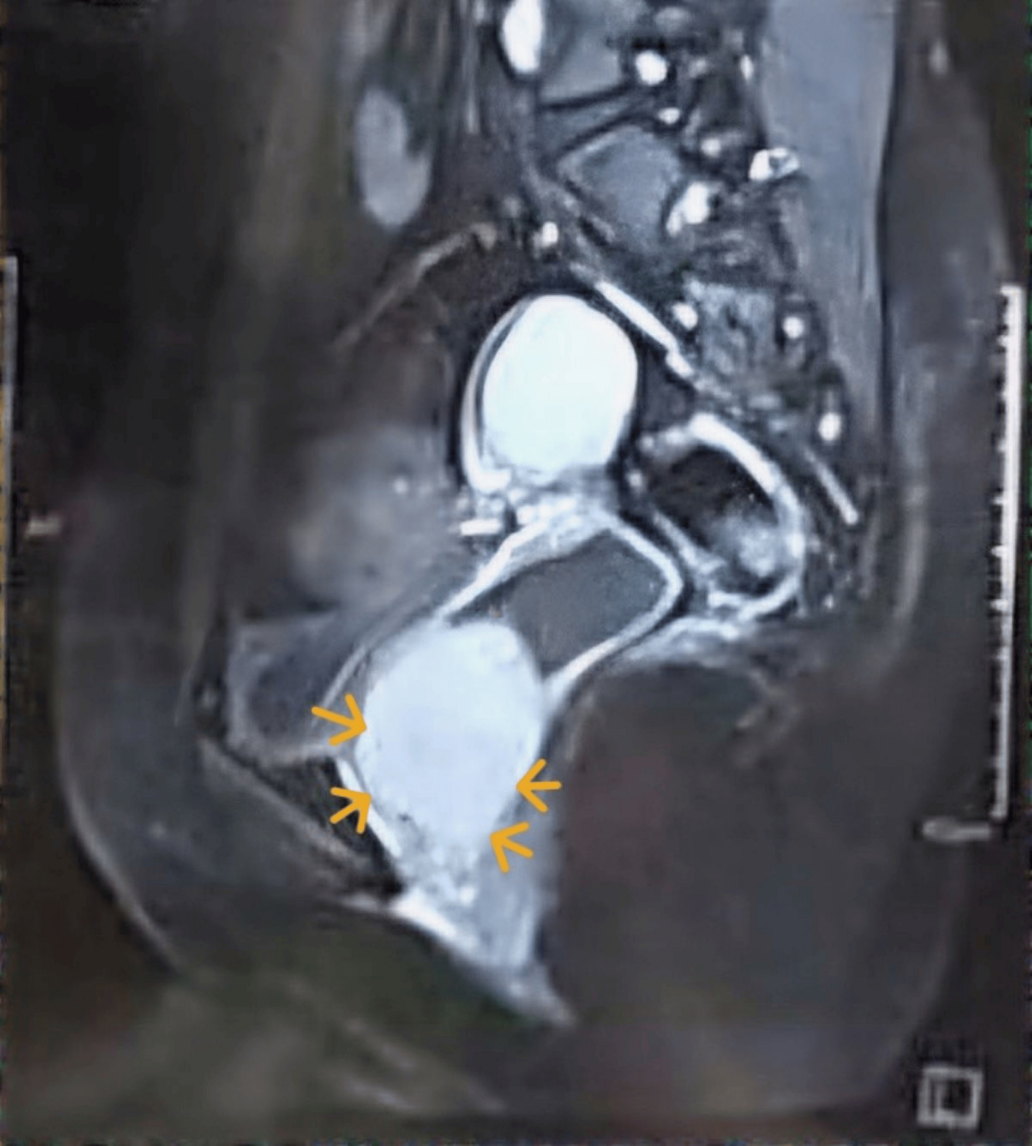
Magnetic resonance imaging with longitudinal view of the pelvis and perineum showing a hyperintense homogeneous mass arising from the anterior wall of vagina (marked by arrow)

T1 sagittal fat-suppressed, post-contrast MRI detected a well-defined solitary, homogeneous lesion of 6x6.7x5.4 cm arising from the anterior wall of the vagina and distending the upper vaginal wall. The intense contrast enhancement was suggestive of a soft tissue mass with vascularity. Uterus bilateral ovaries and cervix were normal. The mass was separate from the uterus and bladder (Figure 2).

The provisional diagnosis considered was a benign vascular mass arising from the anterior wall of the vagina with a differential diagnosis of vaginal hemangioma, lymphangioma, angiomatosis, arteriovenous malformation, leiomyoma, or angiofibroma. An early procedure of total excision of the mass with histopathological evaluation was planned in view of moderate vaginal bleeding with acute retention of urine. 

**Figure 3:**
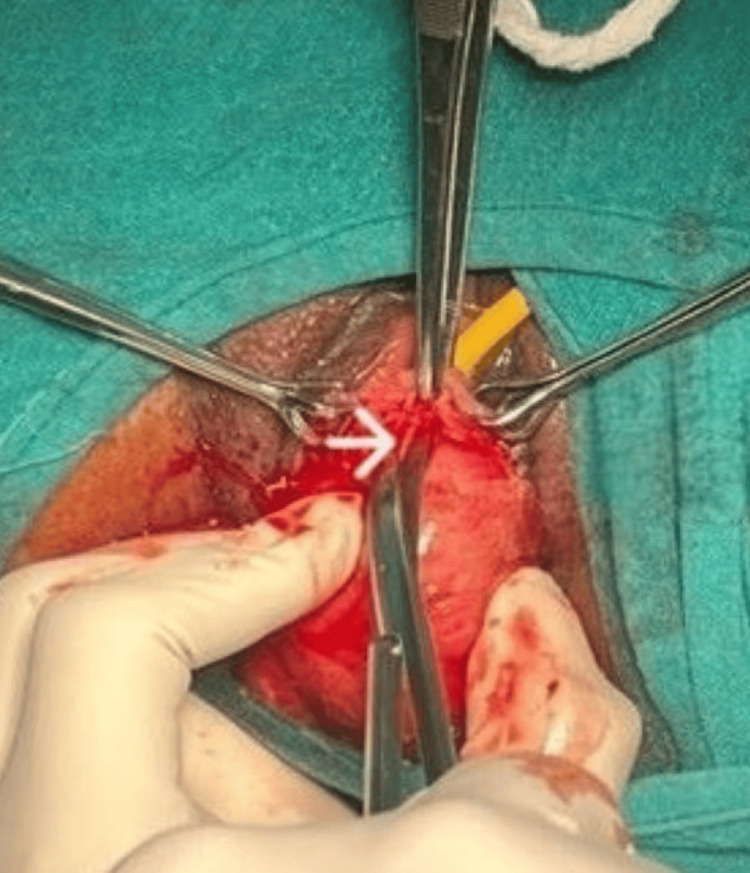
A linear incision was given over the surface of the mass (marked by arrow) and it was enucleated completely.

A linear incision was given over the surface of the mass after injecting adrenaline 1:200000 dilution in the vaginal submucosa to create an avascular plane of dissection. The mass was excised completely, protecting the proximal urethra and bladder (Figure 3).

**Figure 4:**
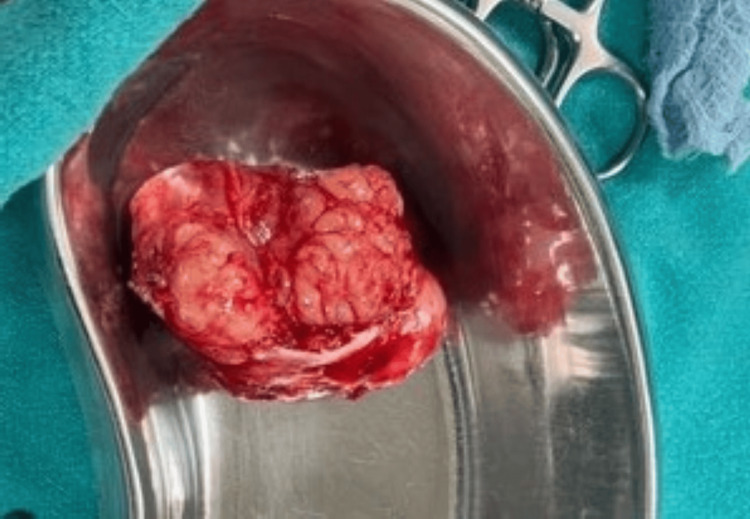
Gross appearance of the excised mass measuring 5x4.6 cm

Gross examination was suggestive of a solid, solitary pinkish-white mass measuring 5x4.6 cm with a lobulated surface (Figure 4).

**Figure 5:**
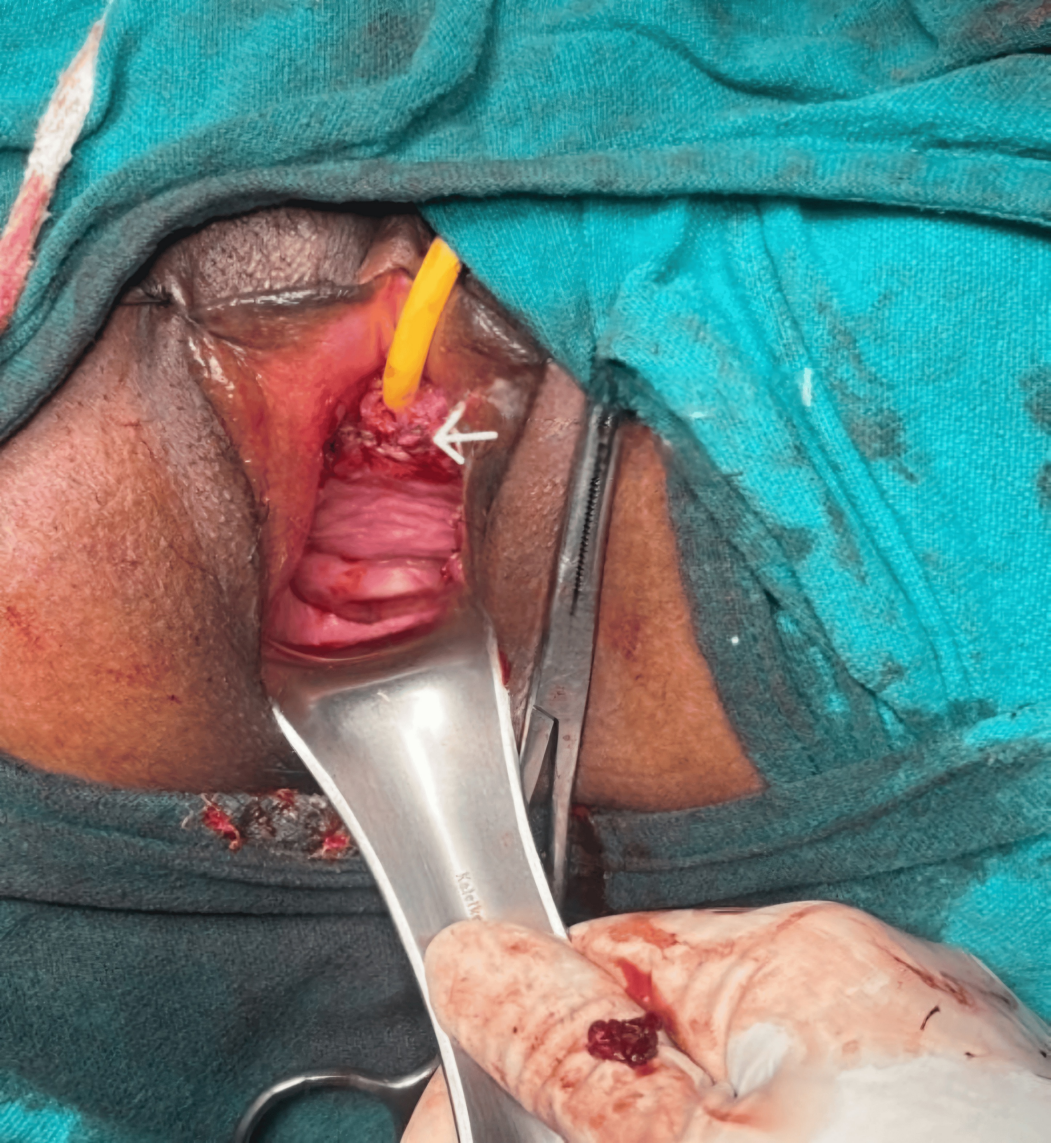
Post vaginal myomectomy the vaginal defect was repaired, achieving good hemostasis (marked by arrow).

The dead space was obliterated with 2-0 Vicryl suture and vaginal flaps were apposed, achieving good hemostasis (Figure 5).

**Figure 6:**
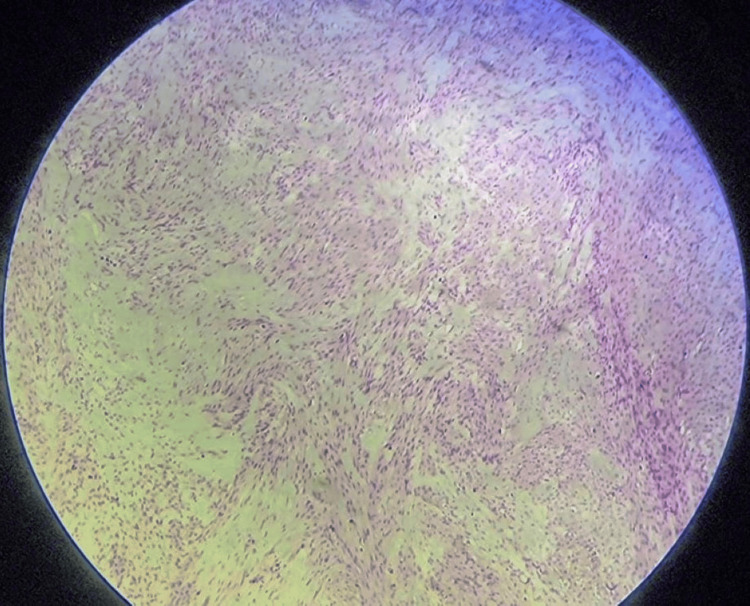
40 X magnification of the haematoxylin and eosin staining histopathological picture of the vaginal leiomyoma

A frozen section of the excised mass was reported as benign leiomyoma of the vagina. The patient was discharged in a stable condition after 48 hours. The Foley catheter was removed a week after the surgery without any voiding difficulty in the patient. Post-surgery, the patient recovered well with good repair and healing of the vaginal wound. The final histopathology report was suggestive of benign leiomyoma of the vagina with no evidence of malignancy (Figure 6).

## Discussion

Vaginal leiomyomas are extremely rare benign tumors of the female genital tract, usually affecting women between 35-50 years of age [1]. They can arise from the anterior, lateral, or posterior wall of the vagina, the most common site being the anterior vaginal wall in the midline, as was seen in our case [2]. These tumors most likely originate from embryonic cells, vascular smooth muscles, or smooth muscles of the vagina, rectum, bladder, and urethra [3]. The tumor is usually solitary, rounded, well-circumscribed, mobile, non-tender, slowly growing, and may or may not be pedunculated [2]. The consistency may be solid or cystic depending on the degenerative changes like hyalinization, liquefaction, calcification, necrosis, and cystic degeneration [4]. Although these tumors are usually slow-growing and benign, local recurrence after incomplete removal and sarcomatous changes can occur [5].

The presenting symptom of vaginal leiomyoma varies depending on the size of the tumor and its site of origin- most vaginal leiomyomas are asymptomatic or present as only a bulging vaginal mass [4]. Occasionally the vagina may be obstructed with the mass resulting in dyspareunia [6]. Anterior wall vaginal leiomyoma with proximity to the urethra and bladder can cause urinary symptoms related to urinary tract infection and obstruction to voiding with acute retention of urine [7], as was seen in our case. When infected, the patient can present with abnormal foul-smelling vaginal discharge, pain, bleeding, or fever and closely mimic a malignant vaginal tumor [8].

The evaluation and diagnosis of vaginal leiomyoma requires a high degree of suspicion, good clinical experience, and imaging modalities like TVS and MRI. These tumors can often be misdiagnosed as uterovaginal prolapse, para-urethral or anterior vaginal wall cyst, bladder or cervical leiomyoma, and vaginal malignancy [5]. MRI is particularly helpful in assessing the site, morphology, and relationship of the tumor with the adjacent viscera. It can confirm the vaginal origin of the mass, which usually appears as solid and well circumscribed with low intensity on T1- and T2-weighted images with a homogeneous contrast enhancement. On the other hand, leiomyosarcoma and other vaginal malignancies show high T2 signal intensity with heterogeneous areas of necrosis and hemorrhage [5]. In our case, MRI could clearly detect the vaginal origin of the mass, distending the upper vagina and clearly separate from the uterus bladder and urethra. The intense contrast enhancement suggested increased vascularity of the mass. 

The treatment of vaginal leiomyoma depends on the site and size of the mass. It usually involves vaginal excision of the mass with repair of the vaginal defect. We injected adrenaline at a 1:200000 dilution in the vaginal submucosa to delineate the boundary of the mass and form an avascular plane for dissection. This also helped to avoid injury to the adjacent bladder and urethra and at the same time, reduced bleeding during enucleation of the leiomyoma and subsequent repair of the vaginal defect. Some authors have similarly used injection oxytocin in the tissue space to create a plane and reduce bleeding during excision or adjuvant therapy with pre-operative gonadotropin-releasing hormone (GnRH) analogs to reduce the size and vascularity of the leiomyoma-prior to its removal [1,9]. For large vascular, vaginal leiomyomas, selective embolization of its vascular supply to reduce the risk of bleeding during myomectomy and vaginal repair has also been described in the literature [10]. Some authors have combined laparoscopic examination of internal genital organs along with vaginal removal of a high posterior wall vaginal leiomyoma [7]. More recently, vaginal myomectomy by transvaginal natural orifice transluminal endoscopic surgery (vNOTES) has been reported to be superior to traditional vaginal myomectomy as it provides a clearer surgical field and better outcome in the patient [11].

Our patient who underwent conventional vaginal myomectomy had an uneventful recovery with good healing of the vaginal wound. Histopathology examination of the excised mass confirmed benign vaginal leiomyoma.

## Conclusions

Vaginal leiomyomas are extremely rare benign tumors of the female genital tract, mostly arising from the anterior vaginal wall in the midline. Large anterior vaginal leiomyomas in close proximity to the bladder and urethra can present with urinary symptoms of difficulty in voiding, including acute retention of urine. MRI is extremely helpful in assessing the site of the tumor, its morphology, including vascularity, and its relationship with the adjacent organs. Thus for the diagnosis of vaginal leiomyoma, MRI should be the imaging modality of choice. The treatment includes total excision of the leiomyoma with repair of the vaginal defect. Use of Injection adrenaline in dilution (1:200000) can help in creating an avascular plane for dissection, enabling easy enucleation of the leiomyoma and a clean surgical field for repair of the vaginal defect. The final diagnosis is always confirmed by histopathological examination of the excised mass.
